# An effective emotion tendency perception model in empathic dialogue

**DOI:** 10.1371/journal.pone.0282926

**Published:** 2023-03-10

**Authors:** Jiancu Chen, Siyuan Yang, Jiang Xiong, Yiping Xiong

**Affiliations:** 1 College of Computer Science and Engineering, Chongqing Three Gorges University, Chongqing, China; 2 College of Computer and Big Data, Fuzhou University, Fuzhou, China; 3 College of Computer Science and Technology, Chongqing University of Posts and Telecommunications, Chongqing, China; Zhejiang University of Technology, CHINA

## Abstract

The effectiveness of open-domain dialogue systems depends heavily on emotion. In dialogue systems, previous models primarily detected emotions by looking for emotional words embedded in sentences. However, they did not precisely quantify the association of all words with emotions, which has led to a certain bias. To overcome this issue, we propose an emotion tendency perception model. The model uses an emotion encoder to accurately quantify the emotional tendencies of all words. Meanwhile, it uses a shared fusion decoder to equip the decoder with the sentiment and semantic capabilities of the encoder. We conducted extensive evaluations on Empathetic Dialogue. Experimental results demonstrate its efficacy. Compared with the state of the art, our approach has distinctive advantages.

## Introduction

Empathy is a complex socio-emotional behavior resulting from emotional and cognitive interactions [1]. Human-computer dialogue aims to investigate how computers understand natural language and strengthen the connection with the user by sensing emotions [2]. It plays a vital role in improving user satisfaction [3–7].

In the existing empathic response studies, [8–12] controlled the generated contents by a specified emotional label. [13–17] proposed various methods for generating empathic responses, which focus on detecting the user’s emotions and generating appropriate responses. [18, 19] perceive implicit emotions through experience or external knowledge. This allows empathic dialogue systems to learn from a bit of history of dialogue about emotional interactions.

**Fig 1:**
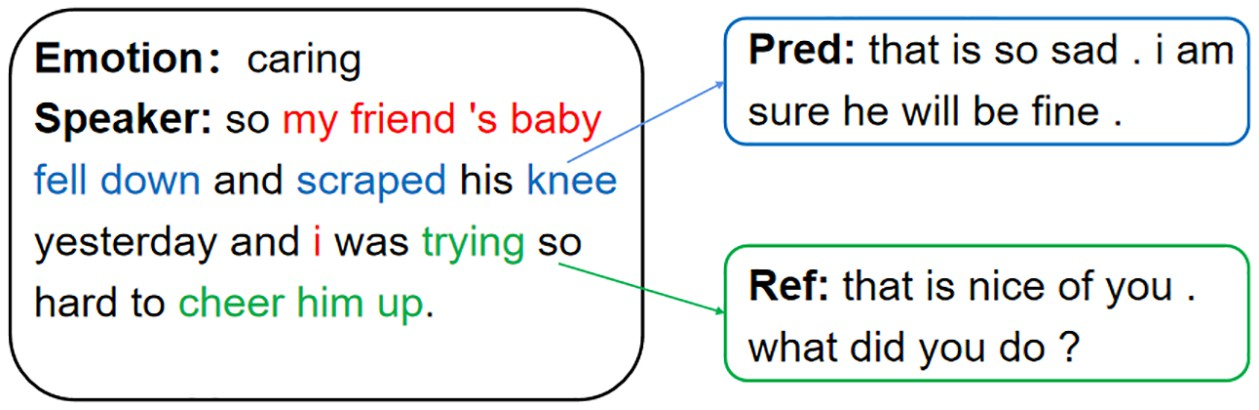
A comparative example from the EMPATHETIC DIALOGUES dataset compares the standard responses with the KEMP model responses.

However, the above emotion detection models overlook the effects of each word on emotion in dialogue. Inspired by the idea of multi-granularity computing [20–22], we note that in an actual multi-round conversation, people can express their emotions through sentiment and non-sentiment words. Therefore, it is crucial for comprehending emotions to perceive the emotions of fine-grained sentences, i.e., words. Fig 1 demonstrates a real-world example of an empathic dialogue. In this illustration, we should respond to the “Speaker” based on his descriptions. “Pred” presents the response created by the KEMP [19] model, an advanced model. However, responding following the “Ref” is more reasonable because it represents the dataset’s default response. Since the KEMP model neglects the latent emotion of many words in the sentence, it has shortcomings in generating empathic replies. Therefore, if we use all words in the dialogue to calculate the intensity value with emotion, we can capture the associations between all words and emotions in the dialogue. Consequently, we can better infer users’ implicit emotions.

To realize this goal, we propose a model of emotional tendency perception for empathic dialogue, named EMO_SA. It quantifies the degree of emotional tendency in sentences to perceive emotions more intuitively, which can generate more empathic responses. EMO_SA consists of the emotion encoder and the shared decoder. First, we evaluate the emotional tendency degree of all words based on a popular taxonomy [13], which classifies emotion into 32 categories. Furthermore, we feed all words’ emotions into a new emotion encoder. In addition, we shared the multi-head self-attention layer weights in the encoder with the decoder and fusion them to enhance the decoder’s ability to respond empathically.

The main contributions of the paper are as follows:

- We propose a novel approach to accurate quantification of the degree of emotion. The approach better captures the user’s emotions based on calculating the similarity of each word in the dialogue with 32 emotional words.
- We propose a shared fusion decoder to mine hierarchical emotions. It fusion the encoder’s ability to perceive emotions and semantics to express empathic responses.
- Experimental results on the empathic dialogue dataset demonstrate the effectiveness of the proposed model. Compared with the current optimal KEMP model, EMO_SA’s accuracy, ppl, distinct-1, and distinct-2 are improved by 0.89, 2.38, 0.23, and 2.29.

The rest of this paper is organized as follows: we introduce related works in Section Related Work. Section Motivation describes motivation of EMO_SA model. Section Method introduces the structure and details of EMO_SA. Experimental results and analysis are provided in Section Experimental Settings. We conclude the whole work and discuss the future work in Section Conclusions and Future Work.

## Related work

**Fig 2:**
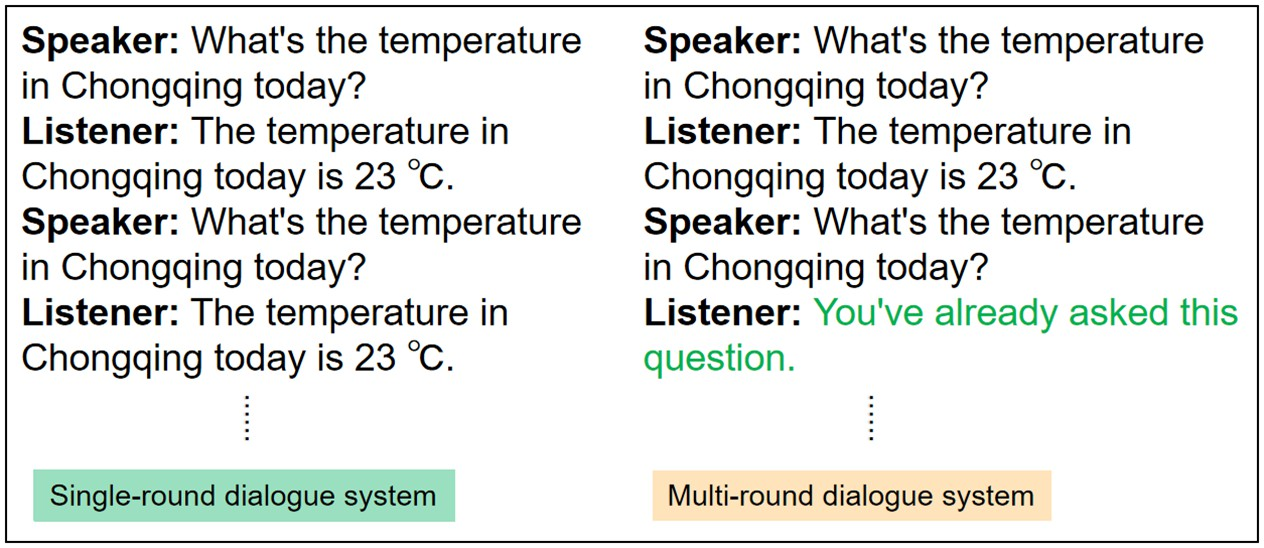
Representation of single-round dialogue and multi-round dialogue tasks.

In the open-domain dialogue system, a single-round dialogue system is often represented as one question and one response, whereas a multi-round dialogue system expresses a user beginning a dialogue or query more than once. The schematic diagram of single-round and multi-round dialogue tasks is shown in Fig 2.

The difference between multi-round and single-round dialogue systems is that the former takes into account historical dialogue content. In previous research on emotional dialogues, Seq2Seq [23] uses an encoder and decoder structure to map the extracted features to the output to solve the problem of indefinitely long speech sequences. But the gradient fading issue can arise. Due to this, Bahdanau et al. [24] proposed an attention mechanism adapted to Seq2Seq, its ability to focus on important information in the encoding during decoding, which facilitates the extraction of semantic features. Zhou et al. [9] proposed an ECM framework based on Seq2Seq, which incorporates an internal dynamic simulation mechanism of emotions and a lexicon-based adaptive response generation mechanism to generate emotional responses. Paper [25] proposed a 25K dialogue dataset based on emotional contexts to facilitate the issue of emotional feelings in human-computer communication. Lin et al. [14] proposed an end-to-end approach to model empathy in dialogue systems: the mixture of empathic listeners (MoEL), which takes into account understanding the user’s emotions and reacts to specific emotions. For the feature that empathic responses will mimic the user’s emotions to varying degrees rather than treating emotions uniformly. Navonil et al. [15] proposed the MIME model, which enhances the contextual correlation of empathy and response. Li et al. [26] proposed to use of coarse-grained dialogue-level and fine-grained token-level emotions to capture the nuances of human emotion, and consider the potential for user feedback to generate more empathetic responses. Sahand Sabour et al. proposed the CEM [27] model by using user emotion recognition [28] and cognitive understanding to enhance the expression of empathy in generative responses. Considering lack of external knowledge would make it difficult for empathic dialogue systems to perceive users’ implicit emotions and to learn emotional interaction problems from limited dialogue history, Qintong Li et al. introduced the NRC-VAD and ConceptNet external knowledge to propose the KEMP [19] model, to understand and express emotions explicitly.

The above researches are good for enhancing empathic responses in dialogue systems, but they do not consider the emotional tendencies of words and cannot perceive emotions accurately. To address this problem, we propose a model for perceiving emotional tendencies for empathic dialogue. By quantifying the sentiment tendency of emotion and non-emotion words in a sentence, we can better perceive the user’s sentiment and generate more empathic responses.

## Motivation

We believe that each word potentially conveys the underlying emotion of the user in the field of human-computer dialogue. In the previous studies, they did not fully utilize the possible sentiment information embedded in each word. Therefore, we propose an emotion encoder to express the correlation between words and emotions. It makes the words reflect the degree of expression for each emotion. We also note that when the encoder extracts information from the input data, the self-attention layer can catch and maintain some semantic information from the original input utterance, which was also overlooked by earlier studies. Therefore, we propose a shared fusion decoder by introducing a shared attention mechanism that enables the attention layer in the decoder and encoder to share part of the semantic information. The parameters of the attention layer are enriched so that the decoder can consider the original information of the input data when generating replies.

## Method

### Overview

**Fig 3:**
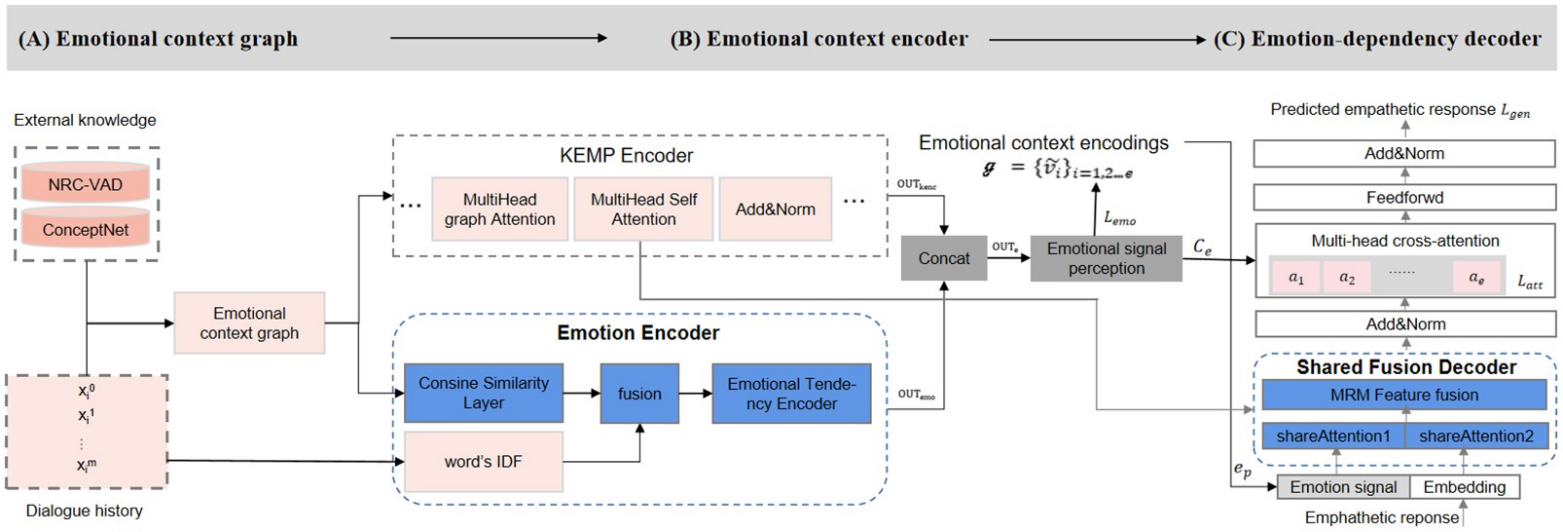
EMO_SA’s architecture diagram. It is composed of an emotional context graph, an emotional context encoder, and an emotion-dependency decoder. Compared with the original KEMP model, we mainly added an emotion encoder of the same level as the KEMP encoder and a shared fusion decoder to the emotion-dependency decoder.

Based on Motivation, we propose the EMO_SA (Emotional_ShareAttertion) model based on KEMP [19], and the overview diagram of the EMO_SA model is shown in Fig 3.

As is shown in Fig 3, we mainly add an emotion encoder and a shared fusion decoder. In summary, we take as input a set of dialogue histories *D* with *B* sequences, i.e. *D* = [*X*_1_, *X*_2_, …, *X*_*i*_, …, *X*_*B*_], where *X*_*i*_ is a sequence containing *m* words and $X_{i}=[x_{i}^{0},x_{i}^{1},...,x_{i}^{m}]$. Through conceptual networks based on the KEMP model, enriched dialogue histories *D* into emotional contexts *g*, and extracted the sentiment signals *c*_*e*_ and *e*_*p*_. Finally, a shared attention network and a transformer-based decoder are used to generate responses *y* = [*y*_1_, *y*_2_, …, *y*_*n*_] with sentiment information.

### Emotional context encoder

Since each word in a dialogue utterance potentially expresses the user’s emotional information, we present an emotion encoder additionally. First, it calculates the emotional correlation for each word with 32 emotion categories, respectively. Then, splice the emotional correlation and the IDF value. Finally, feed it to the emotional tendency encoder to obtain the emotional tendency.

#### Emotional correlation

Each word and emotion in dialogue has emotional correlation. To characterize this correlation, we calculate the cosine similarity for each word vector and the 32 emotion vectors separately.

If the word embedding of the input statement is *w*_*i*_ ∈ [*w*_1_, *w*_2_, …, *w*_*d*_], *d* is the number of words and each emotion vector is *e*_*j*_ ∈ [*e*_1_, *e*_2_, …, *e*_32_]. Then, the emotion correlation of *w*_*i*_ with *e*_*j*_ is,

$$\begin{array}{c} o_{ij}=Cosine(w_{i},e_{j})=\frac{w_{i}\cdot e_{j}}{||w_{i}||\;||e_{j}||}\; \end{array}$$ (1)

During the experiments, we noticed that due to the word embedding layer having certain defects, its calculated values for emotion are small, which cannot reflect the correlation of emotions and interfere with the calculation of motion vectors. In order to significantly represent the correlation of words to emotions, we perform a de-averaging portion by performing the mean value on cosine similarity for each category of emotion, which makes the overall emotion expression of words tend to be stable. That is, we find the mean value of cosine similarity between words and a certain class of emotions *e*_*j*_,

$$\begin{array}{c} Avg(w,e_{j})\;=\;\frac{1}{n}\sum_{i=1}^{n}Cosine({w_{i},e}_{j}) \end{array}$$ (2)

where *n* is the number of words in the whole dataset.

Then perform the de-averaging operation to get the emotional correlation *O*_*ij*_ between *w*_*i*_ and *e*_*j*_,

$$\begin{array}{c} O_{ij}=\;o_{ij}\;-\;Avg(w,e_{j}) \end{array}$$ (3)

#### Word frequency processing

For all words in the dataset, each word has a corresponding word frequency, i.e., the number of occurrences of the word. The example analysis reveals that although high-frequency words like “I”, “you”, and “he” are used frequently in the dialogue, and their emotional significance is not as great; and for other words, such as “like”, “disgust”, “hate”, etc., often express users’ particular emotion. Therefore, in order to reduce the influence of deactivated words and high-frequency words on the judgment of emotional tendency, we introduce the IDF algorithm to distinguish the importance of different words in the dialogue, that is, to get the weight of the word *W*_*i*,*j*_,

$$\begin{array}{c} W_{i,j}=log(\frac{\left|D\right|}{\left|N\right|}) \end{array}$$ (4)

where |*D*| denotes the total number of documents in the corpus, and |*N*| denotes the number of documents containing the word. From Eq 4, it is observed that the weights *W*_*i*,*j*_ are inversely correlated with the frequency of the words appearing in the corpus.

#### Emotional tendency encoder

To obtain the input of the emotional tendency encoder *ET*_*i*,*j*_, we can fuse the de-averaged emotional correlation with the weight information of the words, i.e.

$$\begin{array}{c} {ET}_{i,j}=O_{ij}\times W_{i,j} \end{array}$$ (5)

*ET*_*i*,*j*_ denotes all the emotional tendency of word *i*,*ET*_*i*,*j*_ = [*ET*_*i*,1_, *ET*_*i*,2_…*ET*_*i*,64_]. We input *ET*_*i*,*j*_ into the same emotional tendency encoder as the transformer structure to acquire the output,

$$\begin{array}{c} {OUT}_{emo}\;=\;Emo\_encoder({ET}_{i,j}) \end{array}$$ (6)

where *Emo*_*encoder* denotes the emotional tendency encoder in the emotional encoder.

#### Contact

If the encoder output *OUT*_*kenc*_ of KEMP is expressed as,

$$\begin{array}{c} {OUT}_{kenc}\;=\;KEMP\_encoder(w_{i}) \end{array}$$ (7)

where *KEMP*_*encoder* denotes the encoder in the KEMP model.

We splice *OUT*_*emo*_ with the output of the encoder in KEMP, then the spliced output *OUT*_*e*_ is that,

$$\begin{array}{c} {OUT}_{e}\;=\;{OUT}_{emo}\;⊕\;{OUT}_{kenc} \end{array}$$ (8)

#### Emotional signal perception

Inputting *OUT*_*e*_ into the emotion signal perception is encoded to obtain the emotional context variable g as well as the emotional signal *c*_*e*_, where,

$$\begin{array}{c} g={\left\{\tilde{v_{i}}\right\}}_{i=1,2...k} \end{array}$$ (9)

$\tilde{v_{i}}$ denotes the output of the multi-headed attention layer, and *k* is the number of vertices in the contextual concept network.

$$\begin{array}{c} c_{e}\;=\;\sum_{i\;=\;1}^{m}\frac{exp(\eta_{i})}{\sum_{j\;=\;1}^{e}exp(\eta_{j})}\tilde{v_{i}} \end{array}$$ (10)

*η*_*i*_ denotes the emotional intensity corresponding to $\tilde{v_{i}}$, and *c*_*e*_ is a vector of d-dimensional size.

Then, we use the softmax linear layer to project the vector *c*_*e*_ onto the emotion signal *p*_*e*_,

$$\begin{array}{c} P_{e}(e|g)\;=\;softmax(W_{e}c_{e}) \end{array}$$ (11)

where *W*_*e*_ is a weight matrix of size [32, *d*].

And we use negative log-likelihood estimation as the emotion loss function for parameter learning,

$$\begin{array}{c} L_{emo}\;=\;-log(P_{e}(e=e^{*}|g)) \end{array}$$ (12)

where e* is the actual emotion classification, and *e* denotes the predicted emotion classification.

Finally, we feed the output *c*_*e*_ and *e*_*p*_ semaphores from the emotion context encoder into the emotion-dependency decoder for emotion recognition and generating empathic responses.

### Emotion-dependency decoder

The Emotion-dependency decoder uses the embedding vector from the emotion signal and the standard output as input. The input of the emotion signal is the output *e*_*p*_ of the encoder, while the standard output is passed through the embedding layer to obtain the embedding vector.

Since the self-attention layer of the encoder is able to capture and retain emotional information of the original input utterance when extracting information from the input data. If some semantic information of the encoder is shared with the decoder, it allows the decoder to take into account the original information when generating responses. Therefore, we propose a shared fusion decoder based on the transformer model. It employs two shared attention networks while using a multivariate residual model(MRM) [29] to fuse the output.

#### Multi-headed attention sharing

The multi-head attention-sharing mechanism aims to share the attention information from the encoder to the decoder. We consider that the parameters in the multi-headed self-attention carry certain semantic information, so we share it. That is to say, the parameters of the second self-attention layer in the encoder are shared with the decoder, and they can be expressed as,

$$\begin{array}{c} \begin{array}{c} {}[{MHAtt}_{d1},{MHAtt}_{d2},{MHAtt}_{d3},...,{MHAtt}_{dn}]\;\\ =\;[{MHAtt}_{e2},{MHAtt}_{e2},{MHAtt}_{e2},...,{MHAtt}_{e2}] \end{array} \end{array}$$ (13)

where *MHAtt*_*di*_, *i* = 1, 2, …, *n* denotes the parameter of multi-headed self-attention layers in the encoder, and *MHAtt*_*e*2_ is second level, *n* is the maximum number of layers.

Moreover, to diversify the information in the attention layer of the decoder, we share two self-attention networks with different parameters into the attention layer of the decoder. Then the self-attention $v_{i}^{l}$ of the layer can be expressed as,s

$$\begin{array}{c} v_{i}^{l}\;=\;LayerNorm(v_{i}^{l-1}\;+\;MHAtt(v_{i}^{l-1})) \end{array}$$ (14)

where $v_{i}^{l-1}$ denotes the self-attention of the previous layer, $v_{i}^{0}$ denotes the input from the upper-level structure, i.e., the emotion signal and the word embedding vector of the standard output. And *MHAtt* denotes that it is a multi-headed self-attention sublayer consisting of *H* attention heads, and *LayerNorm* denotes the normalization of the network layer.

The output $v_{i}^{l}$ of the two shared attention networks will be fed into the MRM for feature fusion.

#### MRM feature fusion

For the multi-headed self-attention parameters shared by the encoder, the decoder uses an MRM to fuse the features. The MRM is mainly used to integrate information between different modalities in a multimodal task, and we adapted it to fuse multiple attentional items. The output results of multiple shared attention networks are extracted to fuse the contained emotional information. The MRM is divided into two parts: projection and association.

**Fig 4:**
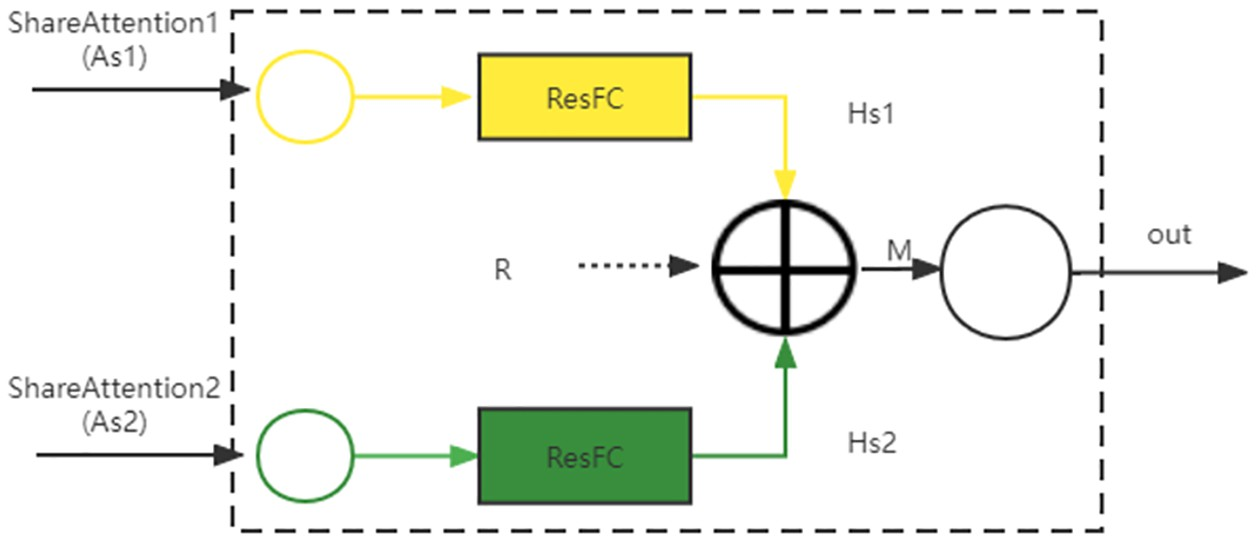
Projection structure diagram.

**Projection**. The projection uses two independent residual networks for the extraction of semantic feature information, and the structure diagram is shown in Fig 4

The projection first maps the features *A*_*s*1_ and *A*_*s*2_ of the two attention layers to the same object space. *A*_*s*1_ maps to *H*_*s*1_ and *A*_*s*2_ maps to *H*_*s*2_. Then,

$$\begin{array}{c} H_{s1}\;=\;A_{s1}\;+\;Relu(W_{ms1}A_{s1}) \end{array}$$ (15)

$$\begin{array}{c} H_{s2}\;=\;A_{s2}\;+\;Relu(W_{ms2}A_{s2}) \end{array}$$ (16)

where *W*_*ms*1_ and *W*_*ms*2_ are the weight matrixs and *Relu* is a nonlinear activation function.

Then the two feature vectors *H*_*s*1_ and *H*_*s*2_ are fused in the same object space. The fused feature vector *H* is,

$$\begin{array}{c} H\;=\;H_{s1}\;⊕\;H_{s2}\; \end{array}$$ (17)

**Fig 5:**
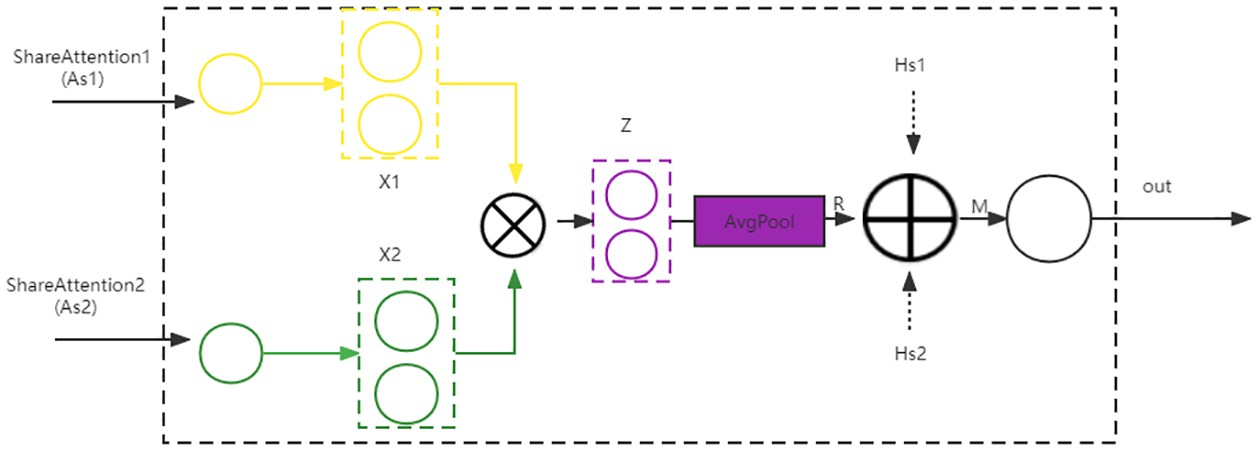
Association structure diagram.

**Association**. The association uses a bilinear strategy to develop feature relationships for different attention. The association structure diagram is shown in Fig 5

As shown in Fig 5, we obtain x1 and x2 by splicing *A*_*s*1_ and *A*_*s*2_ with the weight matrix *W*, respectively, then multiple x1 and x2 to obtain,

$$\begin{array}{c} Z\;=\;A_{s1}^{T}WA_{s2} \end{array}$$ (18)

Since the weight matrix *W* can be decomposed as,

$$\begin{array}{c} W\;=UV^{T} \end{array}$$ (19)

then *Z* can be transformed into,

$$\begin{array}{c} Z\;=\;U^{T}A_{s1}^{T}\;{}^{\circ}\;V^{T}A_{s2} \end{array}$$ (20)

where ° indicates Hadamard accumulation.

In the end, the pooling layer is used to obtain the output *R*,

$$\begin{array}{c} R\;=\;AvgPool(Z) \end{array}$$ (21)

In summary, the output *M* obtained by MRM is the fusion of the two components *R* and *H*, i.e.,

$$\begin{array}{c} M\;=\;R\;⊕\;H \end{array}$$ (22)

This layer takes the outputs *A*_*s*1_ and *A*_*s*2_ of the shared attention layer as input and the vector *M* after fusing the features as output.

Then, we input *M* into the residual and normalization layers to obtain *a*. The obtained *a* is fed into the multi-headed cross-attention network simultaneously with the output *c*_*e*_ from the encoder, which is through the feedforward neural network to obtain the dialogue response $\tilde{y}$. Finally, the final response output *y* is obtained by the normalization layer.

#### Parameter learning

In the emotional encoder, the emotion loss *L*_*emo*_ is obtained by Eq 12, which is designed to improve the correctness of emotion perception. Also, calculating the reinforced emotional attention loss *L*_*att*_ and the response-generated loss *L*_*gen*_. *L*_*att*_ is the emotional attention loss in the KEMP model, which is used to increase the emotional intensity of the responses. And the loss function of response generation is that,

$$\begin{array}{c} L_{gen}=-\sum_{j=1}^{n}logP\left(y_{j}=y_{j}^{*}|y_{1,...,j-1}^{*},g\right) \end{array}$$ (23)

where *y*_*j*_ is the correct result, that is, the response corresponding to the input statement in the dataset, and $y_{j}^{*}$ is the predicted result of the model.

Ultimately, we learn the integrated loss function *L* by adjusting the parameters,

$$\begin{array}{c} L\;=\;\gamma_{1}L_{emo}\;+\;{\gamma_{2}L}_{att}\;+\gamma_{3}L_{gen} \end{array}$$ (24)

where, *γ*_1_, *γ*_2_, and *γ*_3_ are hyperparameters.

## Experimental settings

### Dataset

We use Empathetic Dialogue [13], a benchmark dataset widely used to generate empathic responses, which contains 24,850 multi-round conversations. In each round of the dialogue, the speaker talks about one of the 32 emotions and the content associated with the emotion label, and the listener makes sense of what the speaker says to generate an empathic response [17]. The 32 emotion categories of the Empathetic Dialogue dataset are shown in Table 1.

**Table 1:** Emotional category of Empathetic Dialogue dataset.

Sad	Excited	Furious	Devastated	Anticipating
Afraid	Guilty	Terrified	Impressed	Disgusted
Anxious	Ashamed	Joyful	Nostalgic	Disappointed
Proud	Grateful	Caring	Confident	Apprehensive
Angry	Trusting	Faithful	Annoyed	Embarrassed
Lonely	Hopeful	Jealous	Prepared	Sentimental
Content	Surprised			

**Fig 6:**
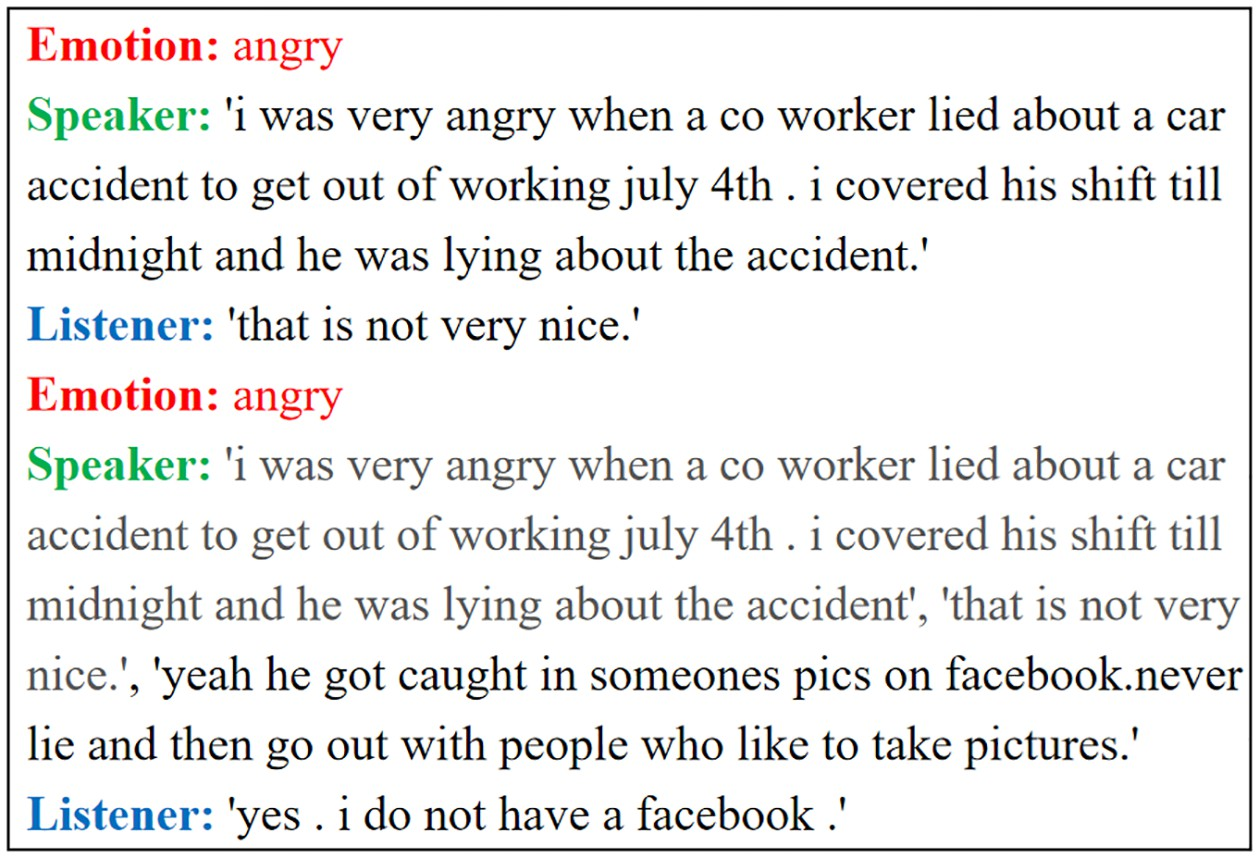
A sample of the Empathetic Dialogue dataset.

A sample of the Empathetic Dialogue dataset is shown in Fig 6. Red denotes emotion, green denotes the speaker’s content, blue denotes the listener’s content, and gray indicates the complete content of the previous dialogue. We can see from Fig 6 that it converts multiple dialogues into a single round of dialogues for processing. That is, it splices the complete dialogues of the previous sequence and uses them as input for the later rounds.

### Baselines for comparison

We compared EMO_SA with the following baseline models:

- The Transformer [30] adopts the encoder-decoder architecture and then uses the self-attention mechanism instead of the RNN network structure commonly used in NLP tasks.
- EmoPrepend-1 [25] is an extension of the Transformer that includes an additional supervised emotion classifier.
- MoEL [14] is a transformer-based generative model that mixes response representations from several decoders and integrates decoder outputs under the projected distribution of emotions.
- MIME [15] is a transformer-based generative model that replicates human emotions based on emotion grouping and uses stochastic sampling for a range of responses.
- EmpDG [16] consists of an adversarial framework including a generator and discriminators that reflect the user feedback, which exploits multi-resolution emotions and user feedback.
- KEMP [19] is an implicit emotion perception model containing external knowledge of NRC-VAD and ConceptNet.

We also conducted an ablation study to better analyze the effects of the different components in our model.

- w/o SA is a model that considers only emotional tendency based on KEMP without considering the shared decoder.
- w/o EMO is a model that considers only the shared decoder on the basis of KEMP without considering feature fusion in the decoder with emotional tendency.
- w/o MRM is a model that considers emotional tendency with the shared decoder but not feature fusion in the shared decoder of the model.

### Evaluation metrics

We used accuracy, perplexity leveL, and distinct-n to evaluate the model.

- Accuracy [31] is the fundamental metric for measuring classification performance, and sentiment accuracy measures the degree of agreement between the sentiment categories in the generated replies and the labels, or the proportion of adequately predicted samples in the classification to the total number of samples.
- Perplexity Level (PPL) [32] is used to evaluate the goodness of the language model, which indicates the confidence level of the model on the candidate response set, and the higher the confidence level, the lower the perplexity level.
- Distinct-n [33] is used to measure the diversity of generated responses. It is not dependent on predetermined responses and can be separated into distinct-1 and distinct-2.

### Implementation details

We partitioned the sentiment dialogue dataset in the ratio of 8:1:1 into a training set, a test set, and a validation set. We use pre-trained Glove vectors to initialize the word embeddings with the same common hyperparameters as the KEMP model. The number of sentiments in the emotion encoder is 32, which is consistent with the categories of emotion in the dataset. The total number of attention layers in the shared attention network is six, and the shared attention layers in the encoder are layer 2 and layer 3. We implemented all models using Pytorch and a single Tesla T4 GPU. In the process of training the model, we found that when each batch contains 16 groups of dialogues and the iteration times of the model are 30,000 times, its performance is optimal. If the number of iterations continues to increase, overfitting will occur. Therefore, the models were trained with 16 dialogues per batch, the number of iterations is about 30,000, and the time is about 5 hours.

### Results and analysis

In the process of replicating KEMP model, we found that when the number of attention layers is 6, the result is the closest approximation to the original KEMP. Therefore, the EMO_SA model we proposed adopted a six-layer attention structure. In order to be fair, in addition to the comparison with the baseline model, we also compared the KEMP_6 model with 6 layers of attention structure in KEMP. The experimental results are displayed in Table 2. The best outcomes from all models are highlighted in bold. We can see that the EMO_SA model has outstanding performance. The accuracy, ppl, distinct-1, and distinct-2 are improved by 0.89, 2.38, 0.23, and 2.29 compared to the integrated optimal KEMP model.

**Table 2:** Automatic evaluation of results.

Models	Accuracy	PPL	Distinct-1	Distinct-2
Transformer	-	37.73	0.47	2.04
EmoPrepend-1	33.28	38.30	0.46	2.08
MoEL	32.00	38.04	0.44	2.10
MIME	34.24	37.09	0.47	1.91
EmpDG	34.31	37.29	0.46	2.02
KEMP	39.31	36.89	0.55	2.29
KEMP_6	37.89	36.05	0.61	3.79
EMO_SA	**40.2**	**34.51**	**0.78**	**4.58**

For the question of how to pick the encoder weights to share with the decoder. In order to reduce the impact of semantic information loss caused by the high number of shared attention layers, we choose the third and lower attention layers for comparison experiment on the basis of without MRM. In the experiment, we compared unshared weights, shared single-layer weights, and spliced different layers weights respectively. The experimental results are shown in Table 3.

**Table 3:** Automatic evaluation results with different layer fusions.

Models	Accuracy	PPL	Distinct-1	Distinct-2
shareAttetion_1	38.72	36.0	0.72	4.39
shareAttetion_2	39.88	35.6	0.72	4.5
shareAttetion_2&3	**40.2**	**34.65**	0.72	4.24
shareAttetion_2&origin	38.07	34.88	0.74	4.31

Where shareAttetion_1 denotes that we just shared the first layer weights, shareAttetion_2 denotes that we just shared the second layer weights, shareAttetion_2&3 indicates that we shared-fused the second and third layer weights, and shareAttetion_2&origin indicates that we shared-fused the second and original layer weights. The best outcomes from all models are highlighted in bold. According to the experimental findings in Table 3, shareAttetion_2&3 performs better in accuracy and perplexity in similar cases of distinct_1 and distinct_2. Therefore, we splice the weights of the second with the third layer in our model.

In addition, we also performed an ablation study to better understand the contributions of the main parts of our model. The results of the ablation study are shown in Table 4.

**Table 4:** Ablation study.

Models	Accuracy	PPL	Distinct-1	Distinct-2
EMO_SA	40.2	34.51	0.78	4.58
w/o SA	39.96	36.037	0.78	4.64
w/o EMO	39.368	35.134	0.61	3.59
w/o MRM	40.2	34.65	0.72	4.24

As shown in Table 4, the accuracy and perplexity of emotions show a significant decrease when we just consider the emotional tendency (w/o SA) based on KEMP. This phenomenon indicates that the shared fusion decoder plays a crucial role in understanding emotions and generating empathic responses. Furthermore, the accuracy, perplexity, distinct-1, and distinct-2 all decrease when we consider the shared fusion decoder (w/o EMO), which demonstrates the importance of emotional tendency. We also consider emotional tendency and shared decoder without fusion (w/o MRM), that the accuracy is same as EMO_SA, but its perplexity, distinct-1, and distinct-2 all deteriorated, which proves fusing is effective.

### Case study

Table 5 compares the responses generated by EMO_SA and the six main baselines. In case 1 EMO_SA generated responses with the most consistent content and accurate sentiment, and it made the responses more empathetic. In case 2–5, EMO_SA perceives the user’s sentiment more accurately and generates responses more empathetic. The above examples demonstrate that EMO_SA can better balance the performance between content and emotion.

**Table 5:** Case study of the generated responses by EMO_SA and the baselines.

**Emotion**	terrified
**Context**	**Speaker**: some time ago, there was a terrible tornado that came through my city.
	**Listener**: it destroyed many homes, a school and some people died.
	**Speaker**: i was so fearful and petrified it would come by our home and destroy it, or harm my family!
**Transformer**	oh no, i am so sorry to hear that.
**EmoPrepend-1**	i am sorry to hear that. i am glad you are not hurt.
**MoEL**	that is awful, i am sorry to hear that. did you have a good time to keep up with them ?
**MIME**	oh no ! i am so sorry to hear that. i hope you can get hurt.
**EmpDG**	oh no ! i am so sorry to hear that. i am glad you had a good family.
**KEMP**	that is horrible. i am so sorry to hear that.
**EMO_SA**	oh no ! i am so sorry to hear that. **i hope you are okay!**
**Ref**	**i am glad you were safe. tornados are not a fun thing** **to experience**.
**Emotion**	terrified
**Context**	**Speaker**: as a kid, i was so frightened any time there was a tornado warning. not sure if it was the wizard of oz effect or what—i just remember being frozen with fear.
	**Listener**: oh, wow ! my son was terrified of tornadoes because of that darn movie, and we do not really even get tornadoes where we live ! did you ever live through a tornado ?
	**Speaker**: that is the thing—no ! i mean, there were some that were kind of close—but nothing like what so many folks have to deal with. i just remember wanting a basement sooooo badly—not something that is common where i grew up !
**Transformer**	i am sorry to hear that. i am sure you will be fine.
**EmoPrepend-1**	i am glad you were not able to get a new one.
**MoEL**	i am not sure what i would like that !
**MIME**	i am glad you are okay !
**EmpDG**	i have never had a i would have cried for a couple of my childhood. i was in a couple of year.
**KEMP**	i would be too ! i would have been so scared.
**EMO_SA**	oh, that is a scary situation. i am glad you are safe !
**Ref**	aw, poor little thing ! how did you overcome this ? or, are you still afraid of them ?
**Emotion**	terrified
**Context**	**Speaker**: one night, i was home alone, when suddenly, i heard a loud bang !
	**Listener**: oh goodness, what was it ?
	**Speaker**: it was a ghost ! i ran into the kitchen to investigate, and i saw what looked like a white dress flapping out the window.
**Transformer**	-
**EmoPrepend-1**	-
**MoEL**	-
**MIME**	oh wow, that is a scary situation. i would be scared too.
**EmpDG**	-
**KEMP**	i would have been terrified !
**EMO_SA**	oh wow, i would have been so scared !
**Ref**	wow, that must have been a terrible experience.
**Emotion**	sad
**Context**	**Speaker**: i was sad when my dad died.
	**Listener**: how long ago did that happen ?
	**Speaker**: it happened 3 weeks ago.
**Transformer**	-
**EmoPrepend-1**	-
**MoEL**	-
**MIME**	i am so sorry. i am sure you will be fine.
**EmpDG**	-
**KEMP**	i am so sorry to hear that. i hope you can get a lot of hard work.
**EMO_SA**	i am so sorry to hear that.
**Ref**	i am so sorry to hear, if you need to talk to someone about it, i am here for you
**Emotion**	content
**Context**	**Speaker**: i cant wait for my new tv that i ordered online and will recive this weekend.
**Transformer**	that is awesome ! i love the show.
**EmoPrepend-1**	that is a good way to be. i am glad you got it.
**MoEL**	that is great. i wish i could have a great time but i loved it.
**MIME**	that is a great feeling. i am sure you will do great !
**EmpDG**	oh wow ! what is your favorite part ?
**KEMP**	that is awesome ! i hope you have a good time.
**EMO_SA**	oh, i love the new i think i would like to go through that.
**Ref**	oh ‥ congrats ‥ you must be happy then

## Conclusions and future work

In this paper, we propose a novel emotional tendency encoder and shared fusion decoder. On the one hand, the emotion tendency encoder measures the emotional tendency of each word underlying the emotions of the user. On the other hand, the shared fusion decoder shares and fuses the self-attention layer of the encoder with the decoder to generate more empathic responses. The experimental results validate the effectiveness of our approach, and the ablation study illustrates the contribution of the main parts of the model. In the future, we will perform further precise mining of the word-emotion relationship to capture users’ emotions more effectively.

## Supporting information

S1 File (ZIP)
